# Age-Related Changes in Ocular Blood Velocity in Suspects with Glaucomatous Optic Disc Appearance. Comparison with Healthy Subjects and Glaucoma Patients

**DOI:** 10.1371/journal.pone.0134357

**Published:** 2015-07-28

**Authors:** Magdalena Asejczyk-Widlicka, Patrycja Krzyzanowska-Berkowska, Beata P. Sander, D. Robert Iskander

**Affiliations:** 1 Department of Optics & Photonics, Wroclaw University of Technology, Wroclaw, Poland; 2 Department of Ophthalmology, Wroclaw Medical University, Wroclaw, Poland; 3 Queensland University of Technology, School of Optometry, Brisbane, Australia; 4 Department of Biomedical Engineering, Wroclaw University of Technology, Wroclaw, Poland; University of Melbourne, AUSTRALIA

## Abstract

**Purpose:**

To evaluate retrobulbar blood flow characteristics of glaucoma suspects with glaucomatous optic disc appearance (GODA) in comparison to healthy control group (CG) and primary open angle glaucoma patients (POAG) and assess the effect of age.

**Methods:**

145 patients from a single glaucoma clinic were enrolled and classified into two diagnostic groups (GODA and POAG). Third group of subjects consisted of 67 age matched individuals (CG). Retrobulbar blood velocity measurement in central retinal artery was performed using color Doppler imaging (CDI). CDI images were processed in custom software leading a range of parameter estimates from a continuous waveform signal. The effect of age on the estimated parameters was evaluated with the stepwise forward regression and ANCOVA in which age was used as a continuous factor. One-way ANOVA was used to test for the differences in the CDI parameters between the three considered groups. Correlation between restive index (RI) and pulsatility index (PI) was assessed with a bilinear fitting guaranteeing no discontinuities in RI intercept estimate. Fisher test was used to assess the applicability of a bilinear PI/RI relationship, while the statistics of the RI intercept estimate were evaluated using the bootstrap.

**Results:**

ANCOVA showed significant interaction between age and group (p<0.05) for five out of nine considered CDI parameters. The RI intercept for CG and GODA groups was 0.602±0.047, and 0.574±0.044 respectively, while the RI intercept of 0.934±0.066 was found for the POAG.

**Conclusions:**

The observed similarity of CG and GODA group and dissimilarity between GODA and POAG groups in terms of PI/RI relationship is remarkable. Age may play some role in the different mechanisms occurring in blood velocity dynamics in GODA and POAG subjects but it is not a strongly determining factor.

## Introduction

Measurement of retrobulbar blood velocity with color Doppler imaging is a powerful noninvasive technique for assessing the eye’s hemodynamics [1–3]. Glaucoma suspects with glaucomatous optic disc appearance (GODA) can be characterized by clinically assessed narrowing of the neuroretinal rim with optic cup concentric enlargement, localized notching, or both, normal visual fields, open angle, and normal levels of intraocular pressure. It is not entirely clear why some of the subjects from the GODA group convert to the primary open angle glaucoma while others remain in stable conditions for a long period of time [4]. Studying ocular blood velocity characteristics could shed some light on this problem as often reduced velocity and increased resistance in ocular blood velocity precede glaucomatous damage [5]. The characteristics of ocular blood velocity may carry some predictive information for the GODA group [6–11]. They could also point out the differences between different subject groups.

Age is an important factor as it affects both the biomechanical parameters of the eye such as the scleral rigidity as well as the retrobulbar hemodynamics [12–14]. However, there are no specific reports dedicated to the age-related changes in suspects with GODA.

The majority of works utilizing color Doppler imaging focus on four major parameters of ocular blood velocity including peak systolic velocity (PSV), end diastolic velocity (EDV), Pourcelot’s resistive index (RI) and pulsatility index (PI). Additionally, parameters such as the ratio between PSV and EDV [1], the mean velocity (MV), the early systolic acceleration (ESA), mean systolic (Sm) and diastolic (Dm) velocities, and the ratio between them have been considered [15].

**Fig 1:**
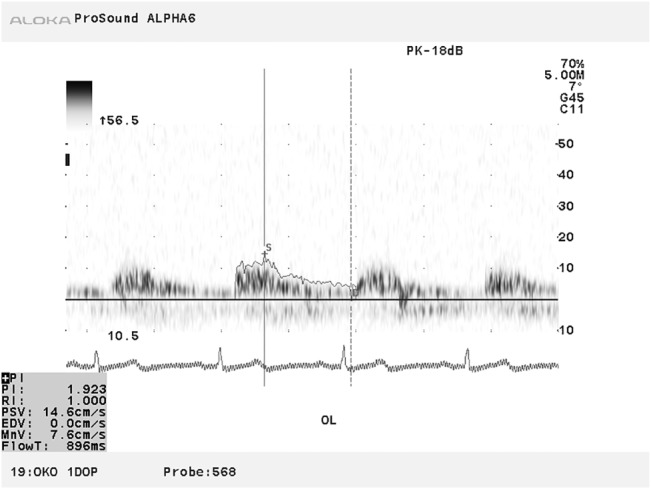
An example of output from the ALOKA CDI with erroneously estimated value of EDV equal to zero and subsequently misrepresented values of PI and RI.

For the ocular blood velocity parameters to have a predictive or discriminative value, they need to be characterized by high repeatability and reproducibility [16–18]. In some color Doppler imaging systems, the operator selects the peak and trough of the measured waveform which could be a laborious task in reproducibility studies [1]. On the other hand, automatically placing a trace on the wave front envelop could result in erroneous readings (see Fig 1).

Ocular blood velocity characteristics are usually assessed in the ophthalmic artery (OA), central retinal artery (CRA) and ciliary arteries (CAs). The CRA waveforms (above the zero line) contain information from a single artery and can be acquired in a reproducible manner if taken posterior to the lamina cribrosa [19]. Also, measuring blood velocity in CRA highlights the differences in the autoregulatory response between glaucoma subjects and healthy controls [20].

This study focused on automatic acquisition of retrobulbar blood velocity parameters in CRA derived from a continuous waveform of several heart cycles to evaluate differences between glaucoma suspects with glaucomatous optic disc appearance, healthy subjects, and primary open angle glaucoma patients and to assess the effect of age.

## Methods

### Subjects

The study had a prospective nature. It comprised of 145 patients (49 males and 95 females) and 67 age matched volunteers (31 males and 36 females). The former group was recruited from the regular patients of the Glaucoma Clinic at the Department of Ophthalmology, Wrocław Medical University. The control group was enrolled from the university staff and their family members who had no ocular and systemic pathologies. Subjects were fully informed of the purpose of the study and all procedures and their requirements. Informed subject consent was obtained before any measurements were taken. Written informed subject consent was obtained before any measurements were taken. The project was approved by the Ethics Committee of the Wroclaw Medical University (KB 481/2009) and adhered to the Tenets of the Declaration of Helsinki. Study exclusion criteria included any systemic disease, history of ocular trauma or eye disease other than glaucoma.

The group of patients was divided into two subgroups of those diagnosed as glaucoma suspects with GODA and those with the primary open angle glaucoma (POAG). GODA diagnosis was determined by clinical assessment including narrowing of the neuroretinal rim with optic cup concentric enlargement, localized notching, or both, but normal visual field, an open angle, and intraocular pressure of less than or equal to 21 mmHg. POAG diagnosis was based on glaucomatous changes in the optic nerve head with corresponding visual field defects and high or normal IOP in the presence of an open angle.

None of the subjects was taking any systemic medications. In the POAG group, patients were taking prostaglandins (42%), beta-blocker drops (35%), carbonic anhydrase inhibitor eye drops (28%) and alpha agonists (15%). Forty one percent of patients were taking medications in the GODA group: prostaglandins (17%), carbonic anhydrase inhibitor eye drops (19%) and beta-blocker drops (5%).

### Measurements

All participants of the study underwent review of general and medical ophthalmological history and best corrected distance and near visual acuity. This was followed by the measurement of central corneal thickness (CCT, PalmScan AP2000 A-Scan Biometer, MicroMedical Devices Inc., Calabasas, CA, USA), measurement of intraocular pressure with Goldman applanation tonometry and dynamic contour tonometry (DCT, Pascal tonometer), measurement of systolic and diastolic blood pressure (M3 Automatic Blood Pressure monitor, Omron, Kyoto, Japan), and measurement of blood velocity in central retinal artery (CRA) with color Doppler imaging device (ALOKA Prosound Alpha 6, Aloka Co., Ltd., Japan). Up to three measurements per subject were acquired. For DCT, output data included estimates of IOP, OPA, and HR. For CDI, output data consisted of the raw images that were processed in a custom written software to lead estimates of PSV, EDV, RI, PI, MV, ESA, areas under the systolic and diastolic part of the waveform (S and D, respectively), and the ratio between them (S/D).

Additionally the glaucoma clinic patients underwent optic nerve head examination with Heidelberg scanning laser ophthalmoscope (HRT 3, Heidelberg Engineering, Heidelberg, Germany) and automated visual field examination (HFA II, SITA standard 24–2; Carl Zeiss Meditec, Inc., Dublin, CA) with dynamic test strategy. Output data from the HRT3 included retinal nerve fiber layer (RNFL) and cup/disk ratio (CDR) while the output of automated visual field examination included the mean defect (MD).

### Data analysis

**Fig 2:**
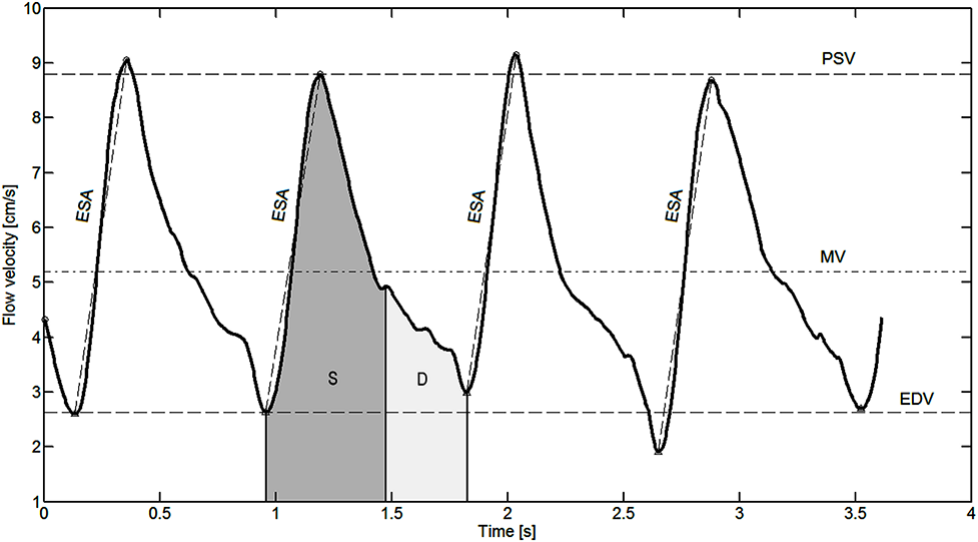
An example of the CDI waveform with robustly estimated parameters.

Raw images, exported from the instrument in a DICOM (Digital Imaging and Communications in Medicine) format were used to extract the CDI waveform signal, that normally consisted of several (up to five) wave pulses. A smoothing filter was applied, each individual pulse was identified using standard peak detection, and a set of parameters PSV, EDV, RI, and PI, MV, ESA, S, D, and S/D was calculated as a median from the several wave pulses (see Fig 2). In this way, a set of robust estimates of CDI parameters was achieved. The differentiation into the systolic and diastolic part of each of the waveform pulses was achieved with an iterative constrained bi-linear least squares fit. For each measurement median values of the above parameters were considered. It is worth noting that rather than using amplitude parameters of the systolic and diastolic section (Sm and Dm) of the waveform [15], we used corresponding energy parameters that amplify the differences in those two phases of ocular blood pulsation.

Following Pinto et al. [21] correlation between RI and PI was assessed. However rather than using piece-wise regression that does not guarantee the linear segments to be connected at the junctions, a bilinear iterative conditional least square fitting was used. In that way, the second line was conditioned on the estimate of the first line and there were no discontinuities in the RI cut point. Additionally, Fisher test was used to test the difference in correlation coefficients between linear and bi-linear fit. A bi-linear fit was assumed only when the Fisher test was rejecting the hypothesis of equal correlation coefficient. The statistics of the RI intercept and the bi-linear slope estimates were evaluated using the bootstrap with a bivariate resampling scheme.

### Statistical analysis

Single factor analysis of variance (ANOVA) was used to test the hypothesis of equal means in GAT IOP, DCT IOP, OPA, HR, CCT, mean systolic and diastolic blood pressures. Student t-test was applied for the RNFL, CDR, and MD as those were compared between GODA and POAG groups. The analyses were preceded with testing for data normality (Jarque-Bera test). The Levene’s test did not reject the hypothesis of equal variance. To test the gender distribution between each pair of groups, the *z*-test for the two proportions was used. For testing the effect of age and group on the CDI parameters, analysis of covariance (ANCOVA) (general linear model, SPSS ver. 22) with age being used a continuous factor was utilized. The significance level for all the tests was set to 0.05.

## Results

The main group demographics together with the group mean GAT IOP, DCT IOP, OPA, HR, CCT, MD, RNFL, CDR, and systolic and diastolic blood pressures are shown in Table 1. Statistically significant differences between all there considered groups were obtained for the gender distribution, GAT IOP, DCT IOP, OPA, and HR. Statistically significant differences were obtained between GODA subject and those of the POAG group in MD, CDR and RNFL (ANOVA-1, *p* < 0.05).

**Table 1:** Number of subjects, mean age, DCT IOP, OPA, HR, CCT, MD, CDR, RNFL, and systolic and diastolic blood pressure for the three considered groups.

Variables	CG	GODA	POAG	*p*
**Number of Subjects (M/F)**	67 (31/36)	38(14/23)	107 (35/72)	0.036
**Mean age (years±SD) (range)**	62±10 (42–79)	61±13 (41–78)	61±10 (41–79)	0.218
**Mean GAT IOP (mmHg±SD) (range)**	14±2 (8–18)	15±2 (12–21)	15 ± 3 (10–31)	<0.001
**Mean DCT IOP (mmHg±SD) (range)**	16±2 (10–21)	17.5±2.1 (9.9–20.5)	17.9 ± 3.3 (7.9–31.9)	<0.001
**Mean OPA (mmHg±SD) (range)**	2.5±1.2 (0.6–6.6)	2.7±1.1 (0.9–5.3)	3.1±1.2 (0.5–6.4)	<0.001
**Mean HR (bpm±SD) (range)**	71 ± 9 (53–94)	73±11 (53–109)	67±10 (43–98)	<0.001
**Mean CCT (μm±SD) (range)**	551±38 (478–640)	546±32 (450–595)	553±38 (424–648)	0.597
**Mean MD±SD (range)**	-	−0.67±1.34 (−3.68–2.20)	−4.05±6.68 (−28.45–2.32)	0.008
**Mean CDR±SD (range)**	-	0.38±0.11 (0.06–0.58)	0.43±0.17 (0.01–0.80)	0.037
**Mean RNFL (mm±SD) (range)**	-	0.23±0.06 (0.06–0.36)	0.19±0.07 (0.01–0.37)	0.011
**Mean Systolic BP (mmHg±SD) (range)**	140±21 (84–197)	149±23 (96–197)	146±22 (88–198)	0.093
**Mean Diastolic BP (mmHg±SD) (range)**	84±10 (59–110)	87±13 (63–126)	88±13 (58–121)	0.105

The group mean and range of the nine considered parameters derived from the blood velocity waveform are collated in Table 2. Statistically significant differences (ANOVA, *p* <0.05) between the three considered groups were obtained for EDV, RI, PI, and MV parameters. ANCOVA results for the CDI parameters, with age set as a continuous factor, are collated in the last column of Table 2. Statistically significant differences were obtained for PSV, EDV, PI, MV, and S.

**Table 2:** Group mean (and range) values of the nine considered CDI parameters. All results for CDI parameters are included in S1 Table.

CDI Parameter	CG	GODA	POAG	ANOVA CG & GODA & POAG *p*	ANCOVA Age & Group *p*
**PSV (cm/s)**	12.29 (7.14–21.78)	12.10 (7.65–21.12)	11.66 (4.47–19.18)	0.084	0.011
**EDV (cm/s)**	4.39 (0.73–13.27)	4.65 (0.45–15.67)	3.50 (0.14–8.79)	<0.001	0.019
**RI**	0.65 (0.37–0.99)	0.63 (0.26–1.04)	0.71 (0.46–1.05)	<0.001	0.079
**PI**	1.03 (0.42–1.92)	1.00 (0.29–2.05)	1.24 (0.62–2.74)	<0.001	0.005
**MV (cm/s)**	7.95 (3.67–18.81)	7.96 (3.87–18.52)	6.98 (2.69–12.44)	0.022	0.001
**ESA**	32.0 (12.9–66.4)	33.7 (17.4–48.8)	33.0 (11.0–61.4)	0.350	0.929
**S (cm** ^**2**^ **)**	4.38 (1.65–14.61)	3.91 (1.51–13.18)	4.20 (1.53–7.93)	0.101	0.006
**D (cm** ^**2**^ **)**	2.23 (0.31–7.78)	1.94 (0.13–10.08)	2.11 (0.16–4.68)	0.193	0.149
**S/D**	2.64 (0.82–15.24)	3.12 (0.76–17.66)	2.57 (0.86–11.98)	0.165	0.055

**Fig 3:**
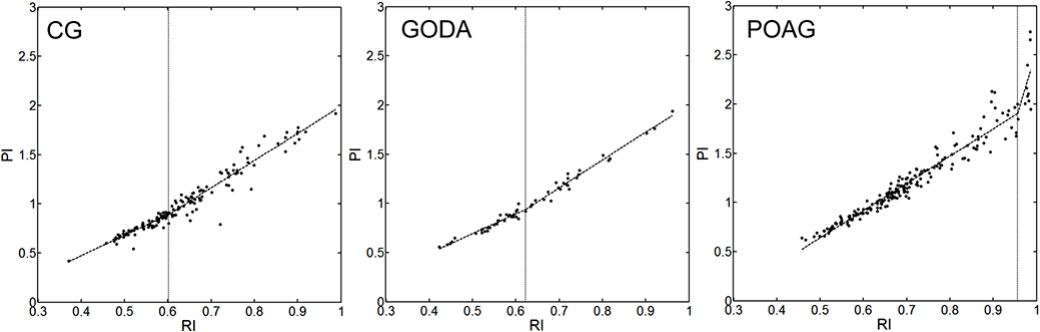
Correlation analysis for RI and PI for three considered groups of subjects. Vertical dotted lines indicate the RI cut points of statistically significant change in the correlation slopes.

Correlation analysis of RI and PI has been considered for the three subject groups (see Table 3). For the control group, statistically significant bilinear trend has been obtained with the RI cut point of 0.602±0.047 (mean±standard deviation evaluated using the bootstrap) and the average slope ratio of 1.416 (slope of 1.995±0.182 and 2.825±0.156 for the first and second line of the bi-linear fit, respectively). The GODA group was characterized by a statistically significant bilinear trend, the RI cut point of 0.574±0.044 and the average slope ratio of 1.570 (slope of 1.733±0.263 and 2.720±0.093 for the first and second line of the bi-linear fit, respectively) achieving similar to the levels to those obtained for the control group. For the POAG group, statistically significant bilinear trend was found with the RI cut point of 0.934±0.066 and the average slope ratio of 3.415 (slope of 2.761±0.120 and 9.430±1.860 for the first and second line of the bi-linear fit, respectively). Fig 3 shows the results of correlating RI with PI for all considered groups of subjects.

**Table 3:** The results of correlation analysis of RI and PI for the three subject groups (CG, GODA, and POAG). The Fisher test was used to determine whether a bi-linear fit was warranted. The statistics of the RI intercept and the bi-linear slope estimates were evaluated using the bootstrap with a bivariate resampling scheme.

	Intercept	*p* (Fisher test)	Slope before intercept	Slope after intercept
**CG**	0.602±0.047	<0.001	1.995±0.182	2.825±0.156
**GODA**	0.574±0.044	<0.001	1.733±0.263	2.720±0.093
**POAG**	0.934±0.066	<0.001	2.761±0.120	9.430±1.860

Correlation analysis was performed between RI and other parameters of ocular blood velocity including MV, ESA, S, D, and S/D. Significant bilinear relationships were obtained for several pair of parameters but results showed at most moderate correlation. Similarly, correlation analysis was performed for all CDI parameters and MD, CDR and RNFL for the glaucoma clinic patients. Weak or moderate correlations were observed but some were statistically significant (indicated in bold font in Table 4).

**Table 4:** The results of the Spearman Rang Order Correlations between CDI parameters and MD, CDR and RNFL. Statistically significant results (*p* < 0.05) are shown in bold font.

		Spearmen’s Correlation Coefficient								
		PSV	EDV	RI	PI	MV	ESA	S	D	S/D
GODA	MD	**0.534**	**0.504**	−0.160	−0.096	**0.620**	0.114	**0.625**	0.087	−0.050
	CDR	−**0.522**	−0.317	−0.099	−0.183	**−0.446**	**−0.356**	**−0.430**	**−0.427**	0.329
	RNFL	0.201	**0.378**	−0.125	−0.093	0.241	−0.076	0.183	**0.466**	**−0.417**
POAG	MD	**0.267**	−0.040	0.084	0.1063	0.127	**0.213**	**0.196**	**0.179**	**−0.235**
	CDR	−**0.334**	0.033	**−0.190**	**−0.201**	**−0.161**	**−0.342**	**−0.234**	−0.100	−0.077
	RNFL	**0.240**	−0.096	**0.200**	**0.225**	0.054	0.140	**0.281**	0.082	0.079

## Discussion

The results of this study demonstrate that age may play some role in the estimated parameters of retrobulbar ocular blood velocity, measured using an automated acquisition of continuous color Doppler imaging (CDI) waveform, but it is not a strongly determining factor. They also indicate potential for the onset and progression of glaucomatous optic neuropathy (GON). Compared with a group of age-matched healthy control subjects, the glaucoma suspects and the POAG patients showed reduced retinal blood velocity and more pulsatile blood velocity in retinal arteries. To our knowledge, this is the first study to examine the influence of age upon ocular hemodynamics including GODA subjects.

The exact mechanism driving these changes is unclear, however, a reduction in the vessel diameter, an increase in peripheral vascular resistance or loss of autoregulatory processes that are strongly influenced by the endothelium have been suggested as likely causes of reduced blood velocity associated with the development of glaucomatous optic nerve damage and age [22–24]. Previous clinical and experimental studies demonstrated that the combination of multiple factors, including structural and functional changes in the microvasculature, blood pressure, IOP and age have a certain impact on retinal and retrobulbar blood velocity [25–29].

A number of previous CDI studies found reduced peak systolic (PSV) and end-diastolic (EDV) velocities, and increased resistivity index (RI) in retrobulbar vessels of glaucoma patients compared to healthy normal controls [30–32]. Interestingly, in our group of patients only the three major parameters of CDI (i.e., EDV, RI and PI) calculated using robust estimators were found to be different between subjects groups, while PSV was not statistically different. Our findings are consistent with report by Rosengarten et al. [33], who found that end-diastolic velocity of the central retinal artery is more sensitive to changes in hemodynamics than is PSV. End-diastolic velocity seems to be more important to the maintenance of downstream blood velocity than is PSV, which is more affected by dynamic blood velocity. Moreover, as EDV is an indicator for resistive changes, it may act as a sensitive marker for vasoreactivity [34]. Furthermore, impendence indices (RI and PI) yield important information on decreased vascular compliance and resistance downstream from the measuring location, which could at least in part be dependent on the degree of arterial stiffness. Variables such as PI and RI have been used extensively in the other fields of medicine such as cardiology and neurology to study downstream resistance. The RI is particularly important in studying vasoreactivity, as it predominantly reflects arterial compliance and pulsatility, while the PI is used for assessment of distal resistance and perfusion pressure and it elevates with the increase in flow velocities pulsation just before loss of autoregulation [21, 35]. In addition, the association of lower end-diastolic velocity with higher impendence indices found in our study may indicate that autoregulation related to an imbalance of endothelium-derived vasoregulatory factors (nitric oxide and endothelin-1) cannot maintain a constant blood velocity despite changes in IOP in GODA and glaucoma subjects; this does not happen in the healthy control group.

Other CDI parameters of CRA such as ESA, S, D, S/D although showed some significant differences did not add more information to the exact nature of the perturbation of ocular blood velocity than that already achieved by the four main CDI parameters.

Given that a decrease in EVD was shown to be a sensitive indication of increased downstream impedance, which also leads to increased resistivity indirectly measured by RI and PI indices [36], our results may indicate that changes in the resistance affect the diastolic flow velocity more than the systolic flow and underlie impaired retrobulbar hemodynamic that may lead to onset and/or progression of glaucoma. This may imply that the age should be considered as a co-factor contributing to, and being indicative of ocular vascular compromise [37–40]. Over time the volume of blood in the vascular bed of the eye diminishes mostly due to decrease in a retinal vessel wall’s ability to stretch in response to increased intraluminal pressure. A large scale study provided evidence that the diameter of retinal arterioles decreases by about 2.1 μm for each decade increase in age [41], which would result on average in a 21% resistance increase from the age of 20 to 80 years. Thus, an increase in impedance indices found in our study seems to indicate that the age-related increase in vascular resistance exceeds that of the potential decrease in compliance.

To our knowledge only one study [21], has previously examined the correlation between impendance indices (PI and RI) in the central retinal artery and they reported non-linear relation with the intercept at RI = 0.77 in glaucoma patients, but not in their control group. These authors reported that non-liner relation between RI and PI, independent of individual RI value indicates a limit in the vessels ability to adapt to increased resistance. Contrary to these findings, we found non-linear responses with the average intercept of 0.602 and 0.574 for the control and GODA groups, respectively while the average intercept of 0.934 was found for the POAG group. These intriguing findings may suggest that in the context of glaucoma onset and/or progression, changes in retinal impedance reflect one or more pathogenetic mechanisms such as arteriolosclerosis and disturbed autoregulation, which contribute to determining vascular distensibility.

The reason for the discrepancy between Pinto et al. [21] and our study may be related to different study designs, patient populations, blood velocity quantification techniques, or CDI machines with varied sensitivity. It must be kept in mind that CDI requires an experienced operator to obtain reliable measurements, and the variability and accuracy of blood velocity measurements among other things depend on a visualization of arteries used for the waveform assessment. The tortuous and variable course of the OA, the lack of any spatial relationship to the optic nerve lowers its detectability rate. Similarly, it is very difficult to determine responses from the small diameter vessels such as short posterior ciliary arteries because of a lack of visual landmarks. Therefore, we decided to use the CRA waveforms as they contain information from the single artery, and can be acquired in a reproducible manner if taken posterior to the lamina cribrosa [19, 42]. Additionally, sensitivity of CDI is also influenced by the reproducibility of the analysis method. By using an iterative constrained bi-linear least squares fit instead of amplitude parameters of the systolic and diastolic section of waveform and piece-wise regression method for estimation of correlation between PI and RI indices we were able to amplify the differences between the systolic and diastolic phase of ocular blood pulsation and detect the RI intercept without any discontinuity.

It is widely recognized that blood velocities in the CRA are influenced by IOP and show an inverse relationship with IOP [43, 44]; thus PSV and EDV fall and the impedance indices rise with increase in IOP. In our study, the mean IOP, measured with GAT and DCT, was significantly higher in POAG group that in GODA group and in normal subjects, and this finding is in agreement with pervious results of Realini et al. [45] and Ceruti et al. [46]. Because the mean IOP was not different between glaucoma and GODA groups, it is therefore unlikely that the alternations in retinal blood velocities in patients with glaucoma resulted from the increased IOP. Indeed, altered retinal blood velocity in glaucoma patients suggests that other factors different from IOP may have important roles in the pathogenesis of glaucoma either directly or indirectly.

Similarly, the OPA measured with DCT of the glaucoma patients was significantly different to that of the CG, while GODA patients demonstrated similar to glaucoma subjects’ relation between OPA and blood flow-related parameters; the OPA was not significantly different to normal subjects. This result was consistent to our previous findings [47] but in contrast to Kotecha et al. [48], who achieved significant differences (at the borderline of *p* = 0.05) in the three groups. Moreover, the OPA results showed positive correlation with RI and PI indices and negative relationship with PSV and EVD in glaucoma and GODA patients. These findings are in contrast to previous studies that demonstrated a positive correlation between the OPA and RI in the CRA only in the healthy population [18, 49]. This may be due to differences in sample characteristic and in measurement procedures. Additionally, the exact significance of the OPA and its behavior in various physiologic conditions as well as its relationship with other blood flow-related parameters remains unclear.

While there is general consensus that ocular blood velocity is reduced in glaucoma, the precise relationship among visual field loss, ocular hemodynamics and structural damage remains debatable. Our data are in agreement with data obtained by Yamazaki et al. [50] and Galassi et al. [51], who found lower ocular blood velocities in glaucoma patients with progressive visual field loss compared with those with a stable clinical course. Additionally, Satilmis et al. [52], showed patients with progressive POAG to have EDV of the CRA inversely correlated to the rate of progression of the visual field MD, which is in agreement with our data. These findings may indicate the potential importance of ocular blood flow disturbance in the development and glaucoma progression.

Only few studies evaluated the correlation between the RNFL thickness and ocular hemodynamics and they illustrated a positive correlation between blood flow parameters (PSV and EDV) and the mean RNFL in POAG patients [53–55]. Our results are in line with these studies as they demonstrate the existence of a correlation between decreased EDV in the central retinal artery with the mean RNFL loss in the POAG group. These findings may imply that blood flow from the CRA to the retinal ganglion cells and inner nerve fiber layer is related to structural changes seen in POAG patients. Our GODA group results, however, showed a significant decrease in EDV in the CRA without changes in the mean RNFL, so it is possible that abnormalities in the retinal circulation exist in the pre-glaucoma stage.

The present study demonstrated that assessing differences in ocular hemodynamic parameters using an automated acquisition of a continuous CDI waveform may help in the future to detect patients with the increased risk of developing glaucoma.

## Supporting Information

S1 Table File containing database of CDI parameters for the tree considered group of subjects. (XLSX)
